# Effectiveness of Radiation Shields to Minimize Operator Dose in the Bronchoscopy Suite: A Phantom Study and Clinical Application

**DOI:** 10.3390/jcm14062114

**Published:** 2025-03-20

**Authors:** Hosang Jeon, Dong Woon Kim, Ji Hyeon Joo, Yongkan Ki, Suk-Woong Kang, Won Chul Shin, Seong Hoon Yoon, Yun Seong Kim, Seung Hyun Yong, Hyun Sung Chung, Taehoon Lee, Hee Yun Seol

**Affiliations:** 1 Department of Radiation Oncology, University of Texas Southwestern Medical Center, Dallas, TX 75390, USA; hjeon316@gmail.com; 2 Department of Radiation Oncology, Pusan National University Yangsan Hospital, Yangsan 50612, Republic of Korea; 3 Department of Radiation Oncology, Pusan National University Yangsan Hospital, Pusan National University School of Medicine, Yangsan 50612, Republic of Koreaapex7171@hanmail.net (Y.K.); 4 Department of Orthopedics, Pusan National University Yangsan Hospital, Pusan National University School of Medicine, Yangsan 50612, Republic of Korea; redmaniak@naver.com (S.-W.K.);; 5 Division of Pulmonary and Critical Care Medicine, Pusan National University Yangsan Hospital, Yangsan 50612, Republic of Korea; 6 Division of Pulmonary and Critical Care Medicine, Department of Internal Medicine, Pusan National University Yangsan Hospital, Pusan National University School of Medicine, Yangsan 50612, Republic of Korea; yskim@pusan.ac.kr; 7 Division of Pulmonary and Critical Care Medicine, Department of Internal Medicine, Severance Hospital, Yonsei University College of Medicine, Seoul 03722, Republic of Korea; 8 Division of Pulmonology, Department of Internal Medicine, National Cancer Center, Goyang 10408, Republic of Korea; 9 Department of Internal Medicine, Ulsan University Hospital, University of Ulsan College of Medicine, Ulsan 44033, Republic of Korea

**Keywords:** scattered radiation, radiation shields, bronchoscopist, assistant, phantom study, clinical application

## Abstract

**Background/Objectives**: Fluoroscopy has been widely adopted in interventional pulmonology, as it facilitates real-time visualization of the bronchoscope, endobronchial ultrasound, and biopsy tools during procedures. The purpose of this study was to evaluate the effectiveness of radiation shields in minimizing scattered X-ray dose to the bronchoscopist in a phantom study and to determine the dose of scattered X-ray dose to medical staff with radiation shields in clinical application. **Methods**: An anthropomorphic torso phantom was positioned on the fluoroscopic table between the C-arm X-ray tube and the image detector to mimic bronchoscopic operations. Upper and lower body lead shields were used to examine the effectiveness of radiation shielding. Scatter radiation rates were assessed at a first operator location using real-time dosimeters with and without protective devices. In clinical application, the scattered X-ray dose of the first operator and main assistant was measured using wearable radiation dosimeters during 20 procedures. **Results**: In the phantom study, scattered radiation without shielding was 266.34 ± 8.86 μSv/h (glabella), 483.90 ± 8.01 μSv/h (upper thorax), 143.97 ± 8.20 μSv/h (hypogastrium), and 7.22 ± 0.28 μSv/h (ankle). The combination of upper and lower body lead shields reduced the scattered X-ray dose by 98.7%, 98.3%, 66.2%, and 79.9% at these levels, respectively. In clinical application, mean scattered X-ray dose rates were 0.14 ± 0.05 μSv/procedure (eye), 0.46 ± 0.51 μSv/procedure (chest), 0.67 ± 0.50 μSv/procedure (hypogastrium), and 1.57 ± 2.84 μSv/procedure (assistant’s wrist). **Conclusions**: The combination of radiation shields significantly reduced the scattered X-ray dose at the operator site in the phantom study. The scattered X-ray dose to medical staff during bronchoscopy can be kept at a low level with the aid of a shielding system.

## 1. Introduction

Fluoroscopic guidance plays a crucial role in interventional pulmonology, enabling bronchoscopists to navigate and visualize the bronchoscope, along with diagnostic and therapeutic instruments, during various procedures. While advanced imaging technologies—such as radial probe endobronchial ultrasound (RP-EBUS), virtual bronchoscopy (VB), electromagnetic navigation bronchoscopy (ENB), ultrathin bronchoscopy, robotic bronchoscopy, and cone-beam computed tomography (CBCT)—have enhanced the diagnostic potential for peripheral pulmonary lesions (PPLs), real-time fluoroscopic imaging remains indispensable for ensuring the success of these interventions [1,2,3].

Fluoroscopy can be used to guide and confirm the position of the bronchoscope and other devices during bronchoscopic procedures, especially for biopsy of peripheral pulmonary lesions. The adoption of interventional bronchoscopic fluoroscopy-guided procedures has been increasing, along with an increase in the acknowledgment of peripheral lung findings deserving histopathological diagnosis, for example, nodules and interstitial lung diseases, or transplantation-related abnormalities. Moreover, the increasing use of cone-beam computed tomography (CBCT) and/or augmented fluoroscopy-guided navigation bronchoscopy exacerbates these issues [4]. Thus, the use of fluoroscopy images for bronchoscopy procedures has resulted in increased scattered X-ray doses for medical staff. The scattered X-ray dose of bronchoscopists and assistants is a major worry in the era of fluoroscopy-guided bronchoscopy, which is becoming more common. Bronchoscopists performing fluoroscopy-guided procedures should know the strategy for the “As Low As Reasonably Achievable” (ALARA) principle to avoid potential skin injury and carcinogenic effects of a cumulative scattered X-ray dose.

Several studies have shown that incorporating shielding devices alongside personal radiation protection gear significantly reduces scattered X-ray dose to the healthcare professionals during fluoroscopic procedures [5,6,7,8,9,10,11]. Additionally, a recently published white paper on radiation in interventional pulmonology emphasized the benefits of supplementary shielding, such as glass screens and table skirts, in minimizing scattered radiation [12]. However, fluoroscopy-guided bronchoscopy has distinct characteristics compared to other fluoroscopic procedures like endoscopic retrograde cholangiopancreatography (ERCP), cardiac catheterization, and interventional radiology, as the operator is positioned above the patient’s head. This unique positioning creates a different relationship between the radiation source, scattered radiation, the patient, and the operator. Therefore, it is challenging to apply the same radiation shielding strategies used in other procedures. Despite its clinical significance, there are relatively few studies on the scattered X-ray dose of healthcare professionals during fluoroscopy-guided bronchoscopy and even fewer studies on appropriate radiation shielding strategies [13,14,15,16].

The fluoroscopy-guided bronchoscopic procedure has distinct positioning compared to other fluoroscopy-guided procedures, necessitating unique shielding strategies. We aimed to (1) evaluate the effectiveness of radiation shields to minimize scattered X-ray dose to the bronchoscopist location in a phantom study and (2) determine the scattered X-ray dose to medical staff with radiation shields in clinical application.

## 2. Materials and Methods

### 2.1. An X-Ray Unit and Radiation Shielding Devices

**Figure 1:**
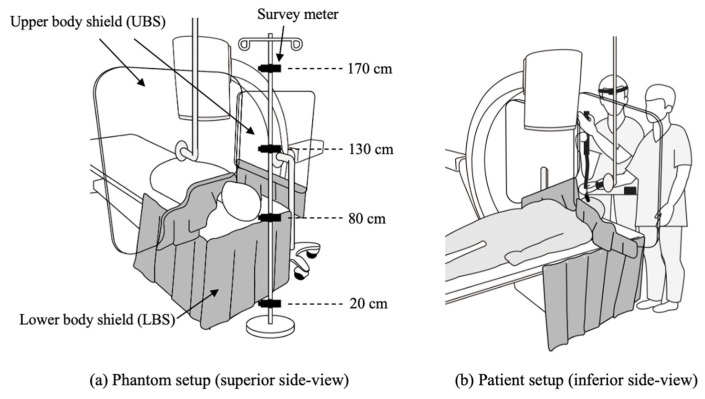
Experimental setup of lead-equivalent plate and a lead-equivalent curtain to shield the operator’s upper and lower body, respectively. (**a**) Superior side view of phantom study and (**b**) inferior side view of clinical application (with operators).

A mobile C-arm X-ray fluoroscopic unit (Cios Select, Siemens Healthineers, Forchheim, Germany) was introduced to irradiate a single phantom and clinical patients. The X-ray tube was located under the couch in all cases. We used a 0.5 mm thick lead-equivalent plate mounted on the ceiling and a rollaway 0.5 mm thick lead-equivalent plate with extension of flexible flaps for the operator’s upper body shield (UBS), as well as a 0.25 mm thick lead-equivalent layered curtain attached around the edge of the couch for the lower body shield (LBS). The overall experimental setup is described in Figure 1.

### 2.2. Phantom Study

An anthropomorphic chest phantom (N1 Lungman, Kyoto Kagaku, Japan) and a head and neck phantom (PH47, Kyoto Kagaku, Japan) were located on the couch to generate scattered radiation that would occur in a real patient. The surface of tube-to-couch distance was 50 cm. A focal point was located 20.2 cm below the surface of the tube. A gamma survey meter (PM1401K-3P, Polimaster, Minsk, Belarus) was used to measure the scattered X-ray dose rate, H*(10), from the operator’s position. The scattered X-ray dose was measured with and without the presence of shielding devices at four different heights, 50 cm cranial from the X-ray tube: glabella (170 cm, close to eye lenses), upper thorax (130 cm), hypogastrium (80 cm, close to the gonads), and ankle (20 cm). The experiments were conducted using four different shielding conditions with and without UBS or LBS, respectively, and all measurements were repeated three times at each height and averaged. The duration of each measurement was at least 10 s, and readings were taken once the values stabilized. Radiation beam conditions were fixed using the tube voltage of 65kVp and the beam current of 4.2 mA. The pulsed fluoroscopy mode with a rate of 30 frames/s was used.

### 2.3. Clinical Application

**Figure 2:**
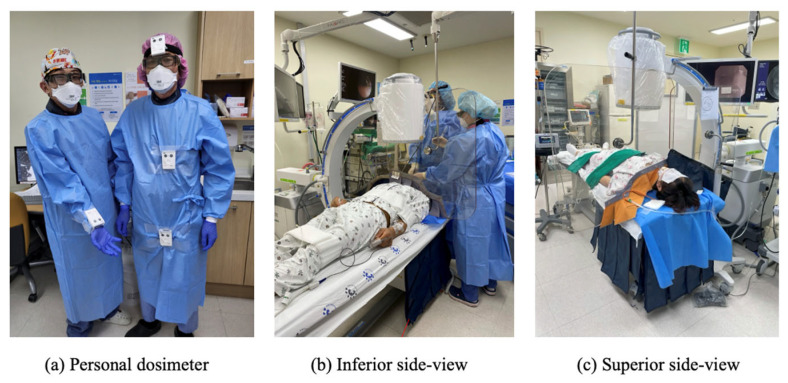
Clinical application setting of lead-equivalent plate for operator’s upper body shield (UBS) and an overlapping lead-equivalent curtain attached around the edge of the couch for operator’s lower body shield (LBS), respectively.

We acquired the scattered X-ray dose rate data of a 177 cm male bronchoscopist and a 173 cm male assistant during the bronchoscopic examinations of 20 cases from April 2023 to June 2023. Although tube voltages and currents were automatically adjusted during the irradiation in the clinical setting, they were close to the beam conditions used for the phantom. Four small personal dosimeters (EPD TrueDose, ThermoFisher Scientific, Waltham, MA, USA) were adopted to measure the scattered X-ray dose rates, Hp(10), of the glabella, upper thorax, and hypogastrium of the bronchoscopist and the right wrist of the assistant. The dosimeters were placed externally to the lead suit, at the bronchoscopist’s upper chest and lower abdomen level. In clinical application, both UBS and LBS were used to protect two medical staff members. The background dose was 0.262 uSv/h in the operation room. The clinical scene of the bronchoscopic examination with the two shields is shown in Figure 2.

### 2.4. Analysis

For the phantom study, we compared the scattered X-ray dose rates of four different measurement sites. The changes in the scattered X-ray dose rates under four different usage conditions of UBS and LBS were also analyzed. The shielding efficacy was defined as the attenuation ratio in percentage (%) between the shielded radiation dose rates and the unshielded dose rate per measurement site. All measurement results were expressed using the mean and standard deviation (SD), and analyzed among four different shielding conditions using Student’s *t*-test via SPSS (Version 19.0, IBM, Armonk, NY, USA). However, for clinical application, the mean scattered X-ray dose rates and their standard deviations with respect to the measurement sites were only analyzed because we used a single shielding condition using both UBS and LBS.

## 3. Results

### 3.1. Phantom Study

**Figure 3:**
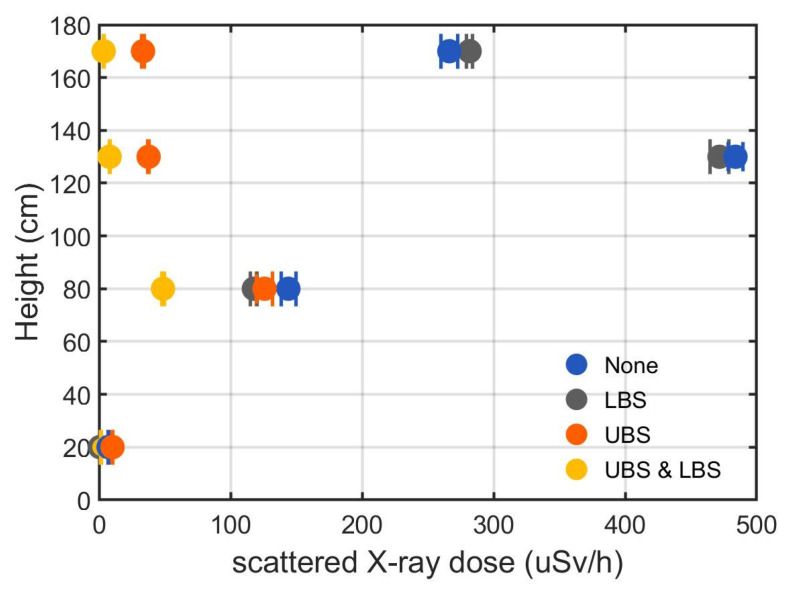
Mean scattered X-ray dose rates and standard deviation with respect to four different shielding conditions. UBS, upper body shield; LBS, lower body shield.

The mean scattered X-ray dose rate without any shields ranged from 7.22 to 483.90 μSv/h for all measurement sites, and the maximum scattered X-ray dose rate was observed in the upper thorax (130 cm height). Scattered X-ray dose rates with LBS alone were similar to those without any shields, except at the ankle (20 cm height), where the absolute value of scattered X-ray dose rate was small. In the case of UBS alone, no significant shield effect was found at lower sites. However, the UBS reduced the scattered X-ray dose rate by an average of 89.8% at higher sites such as the upper thorax and glabella. The use of both UBS and LBS resulted in minimum scattered X-ray dose rates at all sites except at the ankle. All *p*-values computed among different shielding conditions and measurement sites were below 0.01. The mean scattered X-ray dose rates and their standard deviations for each site are listed in Table 1, and are also plotted in Figure 3.

### 3.2. Clinical Application

The 20 bronchoscopic examinations ranged in duration from 19 to 66 min. The mean procedure time was 33.7 min, and the mean fluoroscopy time was 3.8 min. The tube voltage and tube current were adjusted in the range of 60–64 kVp and 1.7–3.7 mA, respectively. The mean scattered X-ray doses and their standard deviations from 20 clinical application were 0.14 ± 0.05 μSv/procedure (2.96 ± 1.91 μSv/h) at the bronchoscopist’s eyes, 0.46 ± 0.51 μSv/procedure (10.1 ± 12.3 μSv/h) at the bronchoscopist’s chest, 0.67 ± 0.50 μSv/procedure (15.2 ± 16.7 μSv/h) at the bronchoscopist’s hypogastrium, and 1.57 ± 2.84 μSv/procedure (24.8 ± 39.3 μSv/h) at the assistant’s wrist. The scattered X-ray doses at the assistant’s wrist were significantly higher than those at the other sites of the bronchoscopist. The magnitudes of mean scattered X-ray dose rates, μSv/h, were smaller than those of their standard deviations for all sites except the eyes. Any proportional relationship was not observed between the fluoroscopy times and the scattered X-ray dose values. The detailed scattered X-ray dose data with their examining conditions for individual cases are presented in Table 2.

## 4. Discussion

The current research indicated that combining extra lead shields minimized the scattered X-ray dose to the medical staff during bronchoscopic procedures. To the best of our knowledge, this is the first study to examine the usefulness of the additional lead shielding device in reducing the scattered X-ray dose during bronchoscopic procedures.

The study utilizing the phantom model revealed that the use of protective shields for both the upper and lower body resulted in minimal scattered X-ray dose levels for the bronchoscopist, except for the ankle area. The scattered radiation measurements in the clinical application were in line with the observed trend in the phantom study, with scattered X-ray dose rates increasing closer to the gap between the UBS and LBS. This demonstrates that the clinical investigation was carried out in a dependable manner. The phantom study showed that when only the LBS was used, the scattered X-ray dose rate increased at the glabella, and when only the upper body shields were used, the scattered radiation increased at the ankle position. Although the absolute value is not very high, results show that shielding either the upper or lower body could increase the scattered X-ray dose to a specific area.

In interventional pulmonology, the scattered X-ray dose for medical staff has been considered lower than that of other fluoroscopy-guided procedures and, as a result, has not been widely studied. However, the adoption of image-guided bronchoscopy has been increasing because of its less-invasive nature [17]; as a result, scattered X-ray dose among bronchoscopists and assistants will increase. To reduce the risk of the stochastic effects, the radiation dose should be as low as reasonably possible while still ensuring that the procedure is effective and efficiently performed [18]. The As Low as Reasonably Achievable (ALARA) principle must be balanced with sufficient image guidance to safely perform these complex procedures without unnecessary scattered X-ray doses. Several factors influence the scattered X-ray dose of medical staff during fluoroscopy-guided procedures. During the interventional procedures, the primary source of scattered X-ray dose for both the operator and the assistant originates from the scatter emanating from the patient, but other scattered X-rays are also emitted by the X-ray tube cover, couch, and shielding devices [19,20,21,22]. There are three main principles for decreasing scattered X-ray dose: time, distance, and shielding [23]. Inevitably, complicated procedures require more time, and it is challenging to control the operator’s distance from the radiation source. However, scattered X-ray dose can be minimized through a better understanding and proper use of protection methods, such as personal protective equipment and radiation protection shields [24].

During bronchoscopy, operators use personal protective equipment such as lead aprons, thyroid shields, and lead eyewear. However, personal protective equipment does not cover the entire body. Moreover, its substantial weight restricts mobility, and it is sometimes avoided due to discomfort. There have been several reports that additionally using a shielding device is effective in reducing scattered X-ray dose during fluoroscopic procedures [5,6,7,8,9,10]. However, even though the unique geometry of the bronchoscope, which is positioned above the patient’s head, allows the operator to place the shield relatively easily without interfering with the procedure, to date, there have been no studies on radiation protection shields in bronchoscopy. Therefore, we conducted a radiation shielding study to establish appropriate strategies to protect medical staff from scattered radiation during bronchoscopy. The present study found that additional shielding devices significantly reduced scattered radiation doses at all locations on the medical staff ’s body. The Radiation Protection for Interventional Pulmonology White Paper recommends, in addition to personal protective equipment, additional shielding such as glass screens and table skirts [12]. Our study provides supporting data for this recommendation and offers practical insights into its implementation in clinical practice.

The International Commission on Radiological Protection (ICRP) recommends that the annual effective dose limit for occupational effective dose should be 20 mSv (20,000 μSv) averaged over five years, with no single year exceeding 50 mSv [25]. In an environment with the use of protective shields for both the upper and lower body, the amount of radiation received during bronchoscopy ranged from 0.14 ± 0.05 to 1.57 ± 2.84 µSv per procedure. When projected over 200 procedures per year, the maximum radiation dose is approximately 880 µSv, which corresponds to 0.88 mSv/year. According to our study, the annual radiation dose when bronchoscopy is performed under sufficiently shielded conditions would be considered low. Using personal dosimeters, the natural radiation dose measured in the bronchoscopy suites over a period of 72 h was 0.262 µSv/h, which equates to an average annual effective dose from all natural sources of about 2.4 mSv.

One of the possible critical issues related to the use of the screens proposed in this work is regarding radiation leakage through gaps in the UBS and LBS close to the patient’s body. The mean occupational radiation dose per bronchoscopy procedure was highest in the assistant’s hand (1.57 ± 2.84 μSv/procedure) in the clinical application and in the hypogastrium region (48.64 ± 1.31 μSv/h) in the phantom study after UBS and LBS. Both were closest to the gap between the UBS and LBS. In the phantom study, it was found that the highest scattered X-ray dose site without shielding was the upper thorax. However, when shielding was utilized, the hypogastrium became the site with the highest radiation scattered X-ray dose and the lowest attenuation capabilities. This is thought to be due to the radiation leakage between UBS and LBS. Additional efforts will be required to reduce radiation leakage through gaps between shields to minimize the dose to the assistant’s hand.

It is crucial to manage the level of scattered X-ray dose for all medical staff participating in the procedure. In this study, scattered X-ray dose was not measured for the positions of nurses other than the bronchoscopist and first assistant. However, scattered X-ray dose is inversely proportional to the square distance from the radiation source. Therefore, considering the minimal amount of scattered X-ray dose measured at the positions of the bronchoscopist and first assistant, it can be assumed that the scattered X-ray dose from the radiation equipment to the nurses standing behind the operator and assistant will be minimal.

This study has several limitations. This was a single-center study, and data were obtained from only a bronchoscopist and assistant. The study was conducted for a relatively short period of 3 months, and the number of procedures was small. However, the geometry used in this study is not significantly different from that of general bronchoscopy procedures, so it is expected to be reproducible. Additionally, although the clinical application were conducted over a short period of time, they included various types of bronchoscopic procedures. Techniques to reduce scattered X-ray dose with conventional X-rays include customized settings for their fluoroscopy system, considering workflow adaptations, and ensuring adequate protection for all throughout the procedure. This study only focused on techniques to ‘optimize protection’. An additional significant factor to consider is the orientation of the dosimeters. In clinical application, the dosimeters affixed to the lower abdomen, chest, and forehead of the primary operator were consistently aligned towards the radiation source. In contrast, the dosimeter attached to the assistant’s wrist may have exhibited variability in orientation due to the movements. Such variability could have contributed to lower recorded levels of scattered X-ray dose. In clinical application, the values measured from the wrist showing a wide range of standard deviation indicate that these concerns are likely to be valid.

Although there may be concerns that additional protective equipment may interfere with patient monitoring, our procedural setting did not experience any interruption in patient monitoring when using a lead acrylic screen. Furthermore, there was no inconvenience in using the monitors of bronchoscopy and fluoroscopy located beyond the lead acrylic screen. If the nurse had to move beyond the lead acrylic screen to inject additional medication, the fluoroscopy was paused briefly and the procedure resumed when the nurse returned. Based on our experience, the use of UBS and LBS was convenient and did not hinder the performance of procedures. No adverse effects for the patient, the staff, or the performance of the bronchoscopy were noted with the use of the shield system.

After the implementation of protective shields, it was determined that the occupational dosage of bronchoscopists was found to be minimal. According to our experimental phase, the lightweight 0.25 mm lead-equivalent aprons and leaded thyroid shields used by the bronchoscopist and assistant during fluoroscopy attenuate 95% of the scattered radiation to the shielded torso. When using protective eyeglasses, they provide shielding equivalent to a lead thickness of 0.75 mm to the lens. Since the dosimeters were attached to the outside of the personal protective equipment, except the assistant’s hands, the level of scattered X-ray dose is further reduced if additional shielding by the personal protective equipment is taken into account. Given the high shielding efficiency of the lead-equivalent aprons, the additional protective effect of radiation shields on the operator’s torso, which is already covered by the apron, may be minimal. However, areas not covered by the apron, such as the hands, arms, legs, and face, can benefit from the extra protection provided by radiation shields. Although the scattered radiation produced during short periods of fluoroscopy may not be considerable in quantity, it accumulates over time. With the anticipated increase in the use of cone-beam CT in the future, the importance of additional radiation shields is expected to grow.

## 5. Conclusions

Our data indicate that the incorporation of additional lead shielding devices may effectively safeguard medical staff from scattered radiation during bronchoscopy procedures. When applied in conjunction with personal protective equipment, this approach significantly reduces scattered X-ray dose to negligible levels. The use of radiation shielding is expected to have a substantial impact on minimizing medical staff’s scattered X-ray dose during bronchoscopy, particularly in challenging cases that involve extended fluoroscopy times. In the execution of interventional bronchoscopy procedures, ensuring the safety of both the procedure itself and the healthcare professionals involved is of paramount importance.

## Figures and Tables

**Table 1:** Mean scattered X-ray dose rates with respect to four different shielding conditions and their shielding effects compared to no-shielding condition (*p* < 0.01 in all cases). Measurements were repeated three times at each height and the readings were taken after at least 10 s when the values stabilized.

Shielding Condition		Height	Scattered X-Ray Dose Rate	Shielding Efficacy
UBS	LBS	(cm)	(μSv/h)	
No	No	170	266.34 ± 8.86	-
		130	483.90 ± 8.01	-
		80	143.97 ± 8.20	-
		20	7.22 ± 0.28	-
Yes	No	170	33.44 ± 1.16	87.4%
		130	37.71 ± 0.73	92.2%
		80	125.84 ± 8.71	12.6%
		20	10.29 ± 0.41	−42.5%
No	Yes	170	281.75 ± 3.41	−5.8%
		130	471.74 ± 10.38	2.5%
		80	117.69 ± 3.68	18.3%
		20	0.68 ± 0.20	90.5%
Yes	Yes	170	3.52 ± 0.27	98.7%
		130	8.05 ± 0.31	98.3%
		80	48.64 ± 1.31	66.2%
		20	1.45 ± 0.38	79.9%

UBS, upper body shield; LBS, lower body shield.

**Table 2:** Scattered X-ray doses of medical staff with examining conditions of 20 bronchoscopic cases in clinical application.

General Information of Examination						Scattered X-Ray Dose (uSv/Procedure)			
ID	Diagnosis	Procedure	Location	Procedure Time (min)	Fluoroscopy Time (min)	Eye (Bronchoscopist)	Chest (Bronchoscopist)	Hypogastrium (Bronchoscopist)	Wrist (Assistant)
1	Squamous cell carcinoma	EBUS-GS	LUL	19	1.6	0.1	0.1	1.7	2.5
2	Bronchial tissue	EBUS-GS	LLL	40	7.1	0.2	0.5	1	1.7
3	A few atypical cell clusters	EBUS-GS	LUL	21	1.6	0.1	0.2	0.2	0.2
4	Adenocarcinoma	ultrathinTBLB	LUL	36	5.9	0.2	0.8	1.5	1.3
5	Vague granulomatous reactions	EBUS-GS	RML	21	2.8	0.2	0.3	1.1	0.4
6	Benign lung tissue	ultrathinTBLB	RLL	28	7.2	0.1	0.3	0.3	0.1
7	Granulomas without necrosis	EBUS-GS	RUL	39	3.2	0.2	2.3	1.6	0.3
8	Chronic inflammation with organizing pneumonia pattern	EBUS-GS	LLL	36	5.5	0.1	0.4	0.4	2.3
9	Chronic inflammation	EBUS-GS	RLL	27	2.8	0.2	0.2	0.2	0.3
10	Chronic inflammation	EBUS-GS	RLL, RML	21	2.2	0.1	1.3	0.3	0.7
11	Invasive mucinous adenocarcinoma	EBUS-GS	LLL	22	2.8	0.1	0.2	0.2	1.3
12	Organizing pneumonia	EBUS-GS	LLL	25	4.6	0.1	0.1	0.4	11.9
13	Foamy histiocytes in lung alveoli	EBUS-GS	LUL	33	5.0	0.1	0.3	0.4	6.4
14	Bronchiolitis obliterans (early CLAD)	TBLC	RLL	66	5.7	0.2	0.4	0.2	0.3
15	Invasive mucinous adenocarcinoma	EBUS-GS	LLL	35	7.3	0.1	0.2	0.52	0.5
16	Adenocarcinoma	EBUS-GS	RLL	25	3.0	0.1	0.3	0.8	0.1
17	Adenocarcinoma	EBUS-GS	RUL	39	1.9	0.2	0.3	0.5	0.2
18	Squamous cell carcinoma	EBUS-GS	RLL	43	4.1	0.1	0.3	1.1	0.7
19	Bronchial tissue	TBLC	RLL	50	0.9	0.1	0.5	0.2	0.1
20	Adenocarcinoma	TBLC	RLL	48	0.9	0.1	0.2	0.8	0.1
Mean				33.7	3.8	0.14	0.46	0.67	1.57
Standard deviation				12.1	2.1	0.05	0.51	0.50	2.84

EBUS-GS, endobronchial ultrasound guide sheath transbronchial lung biopsy; TBLC, transbronchial lung cryobiopsy; ultrathinTBLB, transbronchial biopsy using an ultrathin bronchoscope; LUL, left upper lobe; LLL, left lower lobe; RUL, right upper lobe; RML, right middle lobe; RLL; right lower lobe.

## Data Availability

The data we obtained through our research can be made available upon proper request at any time.

## Abbreviations

The following abbreviations are used in this manuscript:

UBS, upper body shield; LBS, lower body shield; EBUS-GS, endobronchial ultrasound guide sheath transbronchial lung biopsy; TBLC, transbronchial lung cryobiopsy; ultrathinTBLB, transbronchial biopsy using an ultrathin bronchoscope; LUL, left upper lobe; LLL, left lower lobe; RUL, right upper lobe; RML, right middle lobe; RLL; right lower lobe
